# Electrophysiological Correlates of Second-Language Syntactic Processes Are Related to Native and Second Language Distance Regardless of Age of Acquisition

**DOI:** 10.3389/fpsyg.2016.00133

**Published:** 2016-02-12

**Authors:** Begoña Díaz, Kepa Erdocia, Robert F. de Menezes, Jutta L. Mueller, Núria Sebastián-Gallés, Itziar Laka

**Affiliations:** ^1^Center for Brain and Cognition, Department of Technology, Universitat Pompeu FabraBarcelona, Spain; ^2^Department of Linguistics and Basque Studies, Faculty of Arts, University of the Basque CountryVitoria-Gasteiz, Spain; ^3^Department of Neuropsychology, Max Planck Institute for Human Cognitive and Brain SciencesLeipzig, Germany; ^4^Psycho- and Neurolinguistics Group, Institute of Cognitive Science, University of OsnabrückOsnabrück, Germany

**Keywords:** bilingualism, morphosyntax, event-related potentials, P600, age of acquisition, language distance

## Abstract

In the present study, we investigate how early and late L2 learners process L2 grammatical traits that are either present or absent in their native language (L1). Thirteen early (AoA = 4 years old) and 13 late (AoA = 18 years old) Spanish learners of Basque performed a grammatical judgment task on auditory Basque sentences while their event-related brain potentials (ERPs) were recorded. The sentences contained violations of a syntactic property specific to participants' L2, i.e., ergative case, or violations of a syntactic property present in both of the participants' languages, i.e., verb agreement. Two forms of verb agreement were tested: subject agreement, found in participants' L1 and L2, and object agreement, present only in participants' L2. Behaviorally, early bilinguals were more accurate in the judgment task than late L2 learners. Early bilinguals showed native-like ERPs for verb agreement, which differed from the late learners' ERP pattern. Nonetheless, approximation to native-likeness was greater for the subject-verb agreement processing, the type of verb-agreement present in participants' L1, compared to object-verb agreement, the type of verb-agreement present only in participants' L2. For the ergative argument alignment, unique to L2, the two non-native groups showed similar ERP patterns which did not correspond to the natives' ERP pattern. We conclude that non-native syntactic processing approximates native processing for early L2 acquisition and high proficiency levels when the syntactic property is common to the L1 and L2. However, syntactic traits that are not present in the L1 do not rely on native-like processing, despite early AoA and high proficiency.

## Introduction

In a growing global world, learning a second language (L2) has become a socioeconomic need and, consequently, is a mandatory subject in most countries in the world (around 81% of the 119 countries analyzed in (UNESCO World Report: Investing in Cultural Diversity Intercultural Dialogue., 2009). There are three main elements for L2 learners to conquer: phonology (perception and pronunciation), semantics (the meaning of words), and syntax (the structure of the language). Syntax (along with phonology) is an especially difficult aspect for L2 learners to master, while semantics is more easily overcome (Johnson and Newport, 1989; Van Hell and Tokowicz, 2010). One main question in psycholinguistics is whether an L2 is processed through the same neural mechanisms engaged in L1 processing. Event-related potential (ERP) studies have shown clear components indexing L1 syntactic processes and have revealed that approximation to native-like processing of an L2 syntax depends on three main factors: the age of acquisition (AoA), the proficiency achieved, and the similarity between one's first language (L1) and the target L2 (for a review of studies in relation to AoA, proficiency and language distance factors, see Kotz, 2009; Caffarra et al., 2015). Here we aim to investigate how the syntactic similarity between the L1 and L2 influences L2 syntactic processing. We address this question by comparing two groups of non-native listeners that represent the two endpoints of the AoA-proficiency continuum: a typical group of late learners with average proficiency and another group of early learners with native-like proficiency.

Early acquisition favors L2 learning and native-like processing, as shown by the landmark study of Weber-Fox and Neville (1996). They studied Chinese (L1)—English (L2) bilinguals divided into groups according to their L2-AoA: 1–3, 4–6, 7–10, 11–13, and those above the age of 16. Participants performed a visual grammatical judgment task involving semantic violations, as well as three different syntactic violations (phrase structure, specificity constraint, and subjacency constraint). Semantic violations elicited the typical N400 (a negativity around 400 ms) for semantic processing in natives and all non-native groups. For syntactic violations, the ERPs were native-like when the L2 was learned before the age of 11 and not native-like when learned after the age of 11. Natives and early L2 learners (AoA < 11 years old) displayed a left-anterior negativity (LAN) followed by a P600. The LAN is an ERP component elicited by grammatical violations between 300 and 500 ms. The P600 is a posterior positivity starting around 600 ms that indexes syntactic reanalysis and repair. For all those who learned after the age of 11, the LAN was bilaterally distributed. In addition, the P600 was delayed for the 11–13 age group and no P600 was reported for the >16 age group. They concluded that maturational constraints for L2 learning exist, in line with the Critical Period Hypothesis (Lenneberg, 1967), and that native-like neural mechanisms are used for L2 syntactic processing if learned before puberty. After this study, many others have reported non-native ERP patterns for late L2 learners (Hahne and Friederici, 1999; Hahne, 2001; Mueller et al., 2005, 2007; Ojima et al., 2005).

A criticism for the AoA view is that late L2 learners usually achieve lower L2 proficiency than early L2 learners, making it difficult to tell which of the two factors, AoA or proficiency, is the origin of the non-native brain responses (Friederici et al., 2002; Rossi et al., 2006; Kotz et al., 2008). Rossi et al. (2006) recorded the ERP responses of late learners (AoA > 10 years old). Half of the participants were German natives learning Italian, and the other half of the participants were Italian natives learning German. Learners were divided into high and low proficiency groups and submitted to an auditory grammatical judgment task in the L2. High proficiency groups displayed ERP patterns typically found during native language processing: an early left anterior negativity (ELAN) between 100 and 200 ms that is claimed to index syntactic parsing, followed by the syntactic reanalysis and repair P600 response for word category violations, and a LAN-P600 response for subject-verb agreement violations. Low proficiency groups showed a very distinct ERP pattern: they only showed a delayed and reduced P600 in response to both syntactic violations. These results suggested that when late L2 learners attain high proficiency in the L2, they process the L2 in a native-like fashion.

Conversely, other studies have shown non-native ERP patterns during L2 processing despite high proficiency (Ojima et al., 2005; Chen et al., 2007; Dowens et al., 2011; Pakulak and Neville, 2011; Zawiszewski et al., 2011; Erdocia et al., 2014). For instance, Pakulak and Neville (2011) compared the ERP responses to English phrase structure violations of English listeners and late German learners of English (AoA > 10 years old) during an auditory grammatical judgment task. Importantly, the two groups were matched in their English proficiency as measured with a standardized, norm-referenced test and the grammatical judgment task. Despite being equally accurate in the task as the natives, the late L2 learners displayed a non-native ERP response, namely a broadly distributed P600, whereas the natives displayed an anterior negativity followed by a P600. This finding refuted the claim that high proficiency results in native-likeness even when the L2 is learned late in life. Chen et al. (2007) and Ojima et al. (2005) studied the processing of English subject-verb agreement in late learners (AoA = 12 years old) who did not had such a grammatical trait in their native language. Chen et al. (2007) studied Chinese listeners with a visual grammatical judgment task and Ojima et al. (2005) studied Japanese listeners with a visual comprehension task. The processing of verb agreement violations elicited a LAN and a P600 in natives in both studies. However, non-natives did not show any P600 despite being highly proficiency in English, and a LAN was only observed in Ojima et al. (2005). The authors argued that the absence of a similar syntactic structure in the native language of the learners may be responsible for the distinct neural responses between learners and natives. Indeed, studies reporting native-like processing in late L2 learners with high proficiency studied the processing of syntactic features present in both of the participants' languages. For instance, Rossi et al. (2006) studied word category and agreement violations, which are syntactic traits of both of the participants' languages (i.e., German and Italian). Likewise, Kotz et al. (2008) studied phrase structure violations, a syntactic trait also present in both of the participants' languages (i.e., Spanish and English).

Several studies have investigated the role that L1–L2 similarity plays in L2 syntactic processing. These studies consistently show native-likeness (or an approximation) in highly proficient, late bilinguals for syntactic traits that are common to the L1 and the L2 but non-native ERPs for grammatical traits that are unique to the L2 (Tokowicz and MacWhinney, 2005; Osterhout et al., 2006; Sabourin and Stowe, 2008; Dowens et al., 2010; Foucart and Frenck-Mestre, 2011). This results however were not replicated in Tokowicz and MacWhinney (2005) and Aleman Bañon et al. (2014), who reported native-like ERPs for Spanish gender agreement violations in late English learners of Spanish. However, grammatical gender constitutes a highly heterogeneous class across languages encompassing phonological, morphological, lexical, and syntactic phenomena (Corbett, 1991, 1994). Grammatical gender, thus, contrasts with other linguistic traits like word order or ergativity in that no systematic typological correlations are associated to the presence or absence of gender in a given language (Greenberg, 1978). Crucial evidence supporting the major impact that language similarity has on L2 processing comes from studies showing non-native processing even in the most favorable scenario: when native-like proficiency is attained in a frequently used language learned early in life. Zawiszewski et al. (2011) studied the ERP responses elicited by the processing of syntactic traits that were L1-L2 similar or unique to L2 in early Spanish (L1)—Basque (L2) bilinguals (L2 AoA > 3 years old) during a written grammatical judgment task. For the L1–L2 similar condition, i.e., object-verb agreement, a native-like pattern was found (all participants showed a N400-P600 pattern) while a non-native pattern was found for the conditions unique to the L2. For the unique head parameter (subject-object-verb (SOV) and SVO word order) condition, both groups displayed similar P600 effects, but natives showed a left parietal negativity between 300 and 500 ms, while non-natives showed a frontally distributed negativity in the same time window followed by a broad negativity between 500 and 600 ms. For the unique ergative condition, all participants showed a broadly distributed negativity, but only the natives displayed a P600. Erdocia et al. (2014) also reported non-native processing of syntactic traits unique to L2 with the same type of population studied by Zawiszewski et al. (2011), i.e., highly proficient, early Spanish (L1)—Basque (L2) bilinguals. With a written sentence comprehension task, they studied the processing of sentences following the canonical word order of the L2 (SOV) or a non-canonical order (OSV), both of which differed from the canonical L1 word order (SVO). The comparison of the O and S elements in the second position of the sentence elicited a P600 in early bilinguals but a left temporal–posterior negativity between 400 and 550 ms in natives. Thus, Zawiszewski et al. (2011) and Erdocia et al. (2014) showed that, despite early acquisition and high proficiency, the processing of divergent L1–L2 traits involved non-native processing mechanisms.

Overall, findings on L2 syntactic processing suggest that native-like brain mechanisms are only engaged in processing L2 syntactic traits that are present in the L1, while AoA and proficiency seem to influence the efficiency of the native-like brain mechanisms (as measured by the amplitude and latency of the ERPs). Nevertheless, the impact that AoA and proficiency have in the processing of syntactic traits unique to L2 has not been systematically studied. The present study aims to investigate the role of AoA and proficiency in the processing of syntactic traits that diverge in L1 and L2. To the best of our knowledge, this is the first study comparing early and late L2 learners in their processing of syntactic traits that differ in the similarity between L1 and L2 employing the same experimental procedures.

In the present study, 25 early Spanish (L1)—Basque (L2) bilinguals (AoA = 4 years of age) and 25 late L2 learners (AoA > 16 years of age) participated in an auditory grammatical judgment task on Basque (L2) sentences while their EEG signal was recorded. Spanish and Basque are typologically very different and provide the opportunity to compare grammatical traits that are either present or absent in participants' L1 (Spanish). Spanish is a Romance language, while Basque is a language isolate (De Rijk, 2007). Both Spanish and Basque have verb agreement, agreeing in person and number between a verb and its arguments. However, Spanish only agrees with the subject (*1a*), whereas Basque has multipersonal agreement, meaning the verb must agree with subject and object concurrently (*1b*).

1. *a*. Tú me ha**s** visto.       /        *b*. Zu**k** ni ikusi **na**u**zu**.
       You me have-*2.s* seen  /   You-*erg* me-ø*(abs)* seen *1.s*-have-*2.s*
        “You have seen me.”

Spanish and Basque also possess different argument alignment types. Spanish is a nominative-accusative language (like English or German), while Basque has an ergative-absolutive alignment (like Hindi or Georgian). They also have distinct canonical word orders (Spanish is an SVO language, whereas Basque is an SOV language). Basque also differs from Spanish due to its morphological case marking, i.e., the overt marking of the core arguments of the sentence with specific morphemes (see *1b*), a trait not present in Spanish (see *1a*).

In the present auditory grammatical judgment task, half of the sentences heard were grammatically correct, while the other half had either a subject-verb agreement violation, an object-verb agreement violation, or an ergative case violation (see Table 1). Both subject- and object-verb agreement violations presented a mismatch in number between a plural subject or object, respectively, and a corresponding singular agreement marker in the auxiliary verb. Following Zawiszewski et al. (2011), subject- and object-verb agreement are considered as similar in L1 and L2. Note, however, that Spanish, participants' L1, possesses only subject-verb agreement. For the ergative case violation, two noun phrases marked for ergative case were presented in one sentence. Ergative case is unique to L2. In a previous study (Díaz et al., 2011), native Basque listeners were presented with exactly the same task and materials^1^. Native Basque listeners showed a P600 component, an index of syntactic repair and reanalysis processes, in response to these three syntactic violations. In addition, the object-verb agreement violation elicited an early posterior negativity between 150 and 300 ms. The finding of a P600 for subject-verb agreement, object-verb agreement, and ergative case violations is in line with previous studies with native listeners across several languages, such as English, Spanish, Basque, and Hindi (Coulson et al., 1998a,b; Frisch and Schlesewsky, 2005; Nevins et al., 2007; Silva-Pereyra and Carreiras, 2007; Zawiszewski et al., 2011). In addition, in previous studies with Basque and Hindi native listeners, an N400 was found for object-verb agreement and ergative case violations (Nevins et al., 2007; Zawiszewski and Friederici, 2009; Zawiszewski et al., 2011). This N400 effect was interpreted as an index of costs in computing thematic relationships. However, no N400 was found for any of the violations for Basque natives in Díaz et al. (2011) with the same materials used in the present study. Regarding object-verb agreement, the different agreement feature tested in Díaz et al. (2011) and previous studies (Zawiszewski and Friederici, 2009; Zawiszewski et al., 2011), i.e., number vs. person, respectively, could be the reason for the different ERP pattern. It has been suggested that person plays a more salient role in agreement computations than number, based on the finding of larger P600s for person compared to number agreement violations (Nevins et al., 2007; Mancini et al., 2011; Zawiszewski et al., in press). In line with these previous studies, the violation of the person feature in agreement, as compared to number violations, could lead to higher costs in thematic assignments (Díaz et al., 2011). Regarding ergative case violations, the incorrect sentences in Díaz et al. (2011) always had a correct ergative marked noun phrase, whereas in the two previous studies, the case violation sentences did not include a correct ergative NP (Nevins et al., 2007; Zawiszewski et al., 2011). The lack of a correct ergative NP could cause thematic difficulty in assigning the agency of the ergative argument, as reflected by the N400. Thus, the differences in the specific characteristics of the experimental materials between Díaz et al. (2011) and previous studies (Nevins et al., 2007; Zawiszewski and Friederici, 2009; Zawiszewski et al., 2011) could be the cause for the distinct ERP patterns observed.

**Table 1:** **Experimental stimulus examples**.

1. Grammatical Sentence(40 items)
Mikel-en **arreb-ek egunkari-a** saski-a-n ekarri **d-u-te** kiosko-tik
Mikel-[gen.] **sister-the-[erg.pl.] newspaper-the-[abs.sg.]** basket-the-in brought **it-root-they** kiosk-from
*‘Mikel's sisters have brought the newspaper in a* basket *from the kiosk’*
2. Subject–Verb Agreement Violation (40 items)
Mikel-en **arreb-ek** egunkari-a saski-a-n ekarri d-*u kiosko-tik
Mikel-[gen.] **sister-the-[erg.pl.]** newspaper-the-[abs.sg.] basket -the-in brought it-root-*it kiosk-from
3. Ergative Case Violation (40 items)
Mikel-en arreb-ek egunkari-*ek saski-a-n ekarri d-u-te kiosko-tik
Mikel-[gen.] sister-the-[erg.pl.] newspaper-the-*[erg.pl.] basket -the-in brought it- root-they kiosk-from
4. Grammatical Object–Verb Agreement (40 items)
Mikel-en arreb-ek **egunkari-a-k** saski-a-n ekarri **dit-u-zte** kiosko-tik
Mikel-[gen.] sister-the-[erg.pl.] **newspaper-the-[abs.pl.]** basket -the-in brought **them-root-they** kiosk-from
‘*Mikel's sisters have brought the newspapers in a* basket *from the kiosk’*
5. Object–Verb Agreement Violation (40 items)
Mikel-en arreb-ek **egunkari-a-k** saski-a-n ekarri *d-u-te kiosko-tik
Mikel-[gen.] sister-the-[erg.pl.] **newspaper-the-[abs.pl.]** basket -the-in brought *it-root-they kiosk-from

*Bold words represent the critical word for each violation condition from which onset epochs were established*.

In the present study, we expect native-like ERP responses (i.e., a P600 and an additional early negativity for object-verb agreement as in Díaz et al., 2011) in highly proficient, early bilinguals for L2 grammatical traits present in participants' L1 (verb-agreement conditions). In contrast, we expect non-native ERP responses in the same bilingual group for the grammatical trait unique to L2 (ergative case condition). Additionally, the comparison of the results for the two types of verb agreement violations, subject and object, will allow us to investigate whether L2 verb agreement relations are similarly processed. In less proficient, late L2 learners, we expect non-native effects for all conditions. The critical question is whether AoA and proficiency also have an impact on the processing of the unique L2 trait. The unique L2 trait is expected to elicit non-native ERP patterns in both groups of L2 learners. Nevertheless, we expect that highly proficient, early bilinguals will show a different ERP pattern from less proficient, late learners. The differences between the groups for the processing of the unique L2 trait would reveal what the correlates of L2 mastery in non-native ERPs are.

## Materials and methods

### Participants

Fifty healthy adult participants took part in the experiment. All participants were born and grew up in the Basque Country where both Spanish and Basque are spoken. A Basque adaptation of the language history questionnaire from Weber-Fox and Neville (1996) was administered to all participants. This questionnaire assessed the relative use of Spanish and Basque during childhood, adolescence, and at the time of evaluation. In addition, participants rated their own Spanish and Basque proficiency. No participant reported having had auditory, language or neurological problems. All participants were right-handed as assessed with the Edinburgh Handedness Inventory (Oldfield, 1971). All participants signed the corresponding consent form and were paid for their participation. The experiment was approved by the local ethical committee of the University of the Basque Country and followed the American Psychological Association standards in accordance with the Declaration of Helsinki (World Medical Association., 2013).

The group of early bilinguals tested was composed of 25 university students, Spanish-Basque bilinguals (15 female, mean age: 22.68 years old, range 19–30 years old). For all participants, Spanish was the family language from birth to the time of testing. Thus, prior to attending school, participants had just sporadic (if any) contact with Basque. Participants were continuously exposed to Basque from the age of 3 or 4 when starting mandatory bilingual school. Participants were recruited from the University of the Basque Country.

The group of late L2 learners tested was made up of 25 Spanish-Basque bilinguals (17 female, mean age: 26.71 years old, range 19–36 years old). All late L2 learners were Spanish monolinguals who were, at the time and for 2 years prior, attending classroom-based Basque instruction. They were all enrolled in their fourth semester of Basque lessons, thus on their way toward completing a B2 level (Common European Framework of Reference for Languages). They started Basque instruction at a mean age of 24.70 (*SD* = 4.49). Participants were recruited from several *euskaltegi* (official schools dedicated exclusively to teaching Basque to adults) in the Vitoria-Gasteiz area (Basque Country). As all *euskaltegi* centers follow the same curriculum, late L2 learners can be assumed to have the same knowledge of Basque and did not have virtually any contact with Basque language prior to attending Basque lessons. Despite both Spanish and Basque being official languages in the Basque Country, there is a part of the Basque population that has no contact with the Basque language. According to a sociolinguistic survey published by the Basque government (V Encuesta Sociolingüística: 2011 (2003)), 27% of Basque citizens older than 16 are fluent in both Spanish and Basque, 14.7% can understand it but not speak it, and the remaining 58.3% are Spanish monolinguals. All late L2 learners except one had received, or were receiving at the time, higher education (university, college, or apprenticeship studies).

**Figure 1:**
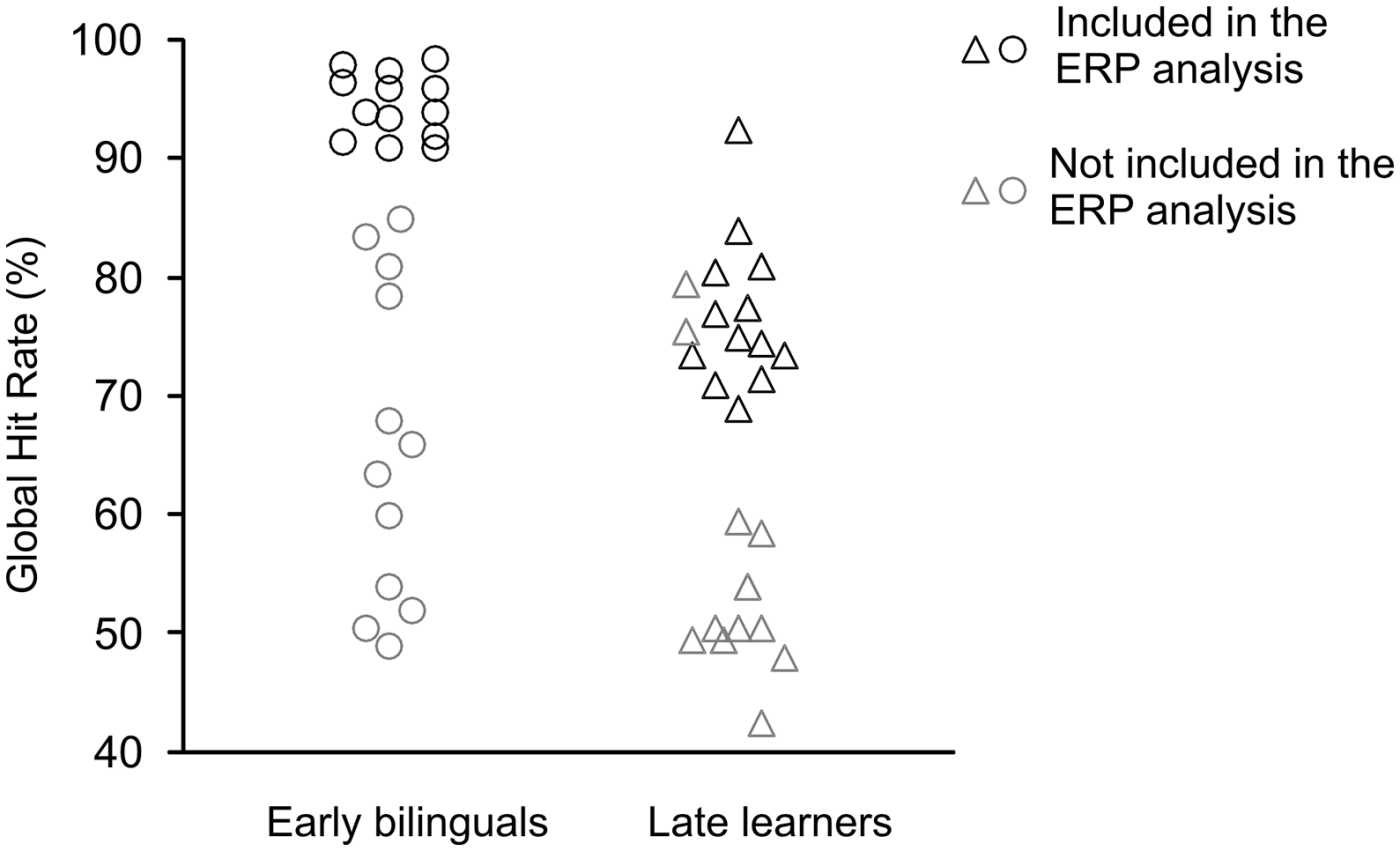
**Participants' global hit rate for early bilinguals and late L2 learners**.

Not all participants were included in the ERP analysis. Late L2 learners' accuracy in the grammatical judgment task varied greatly (see Figure 1, Results) from chance levels (accuracy between 40 and 60%) to relatively good proficiency (accuracy ≥ 69%). Only those late L2 learners with a global accuracy of 69% or above in the task were included in the ERP data analysis. Fifteen late L2 learners had the minimum accuracy required, but due to artifacts in the EEG signal, the data from two of those late L2 learners were excluded from all the analyses. To match the number of participants in each group, 13 early bilinguals with the highest accuracy were included in the ERP analysis. Table 2 shows participants' characteristics and self-reported relative language use through life span for the sample of participants included in the ERP analysis.

**Table 2:** **Group characteristics and self-reported relative use of Spanish and Basque during life span, ranging from 1 (Basque only) to 7 (Spanish only), and self-reported proficiency, ranging from 1 (perfect) to 4 (poor)**.

	**Early bilinguals (*n* = 13)**	**Late L2 learners (*n* = 13)**
Age^*^	23.23 (3.03)	26.76 (5.16)
AoA of Basque^*^	3.23 (0.43)	24.76 (5.16)
Sex (females)	8	10
**RELATIVE LANGUAGE USE**		
**Before school**		
Home	6.30 (1.25)	6.92 (0.27)
**Primary school**		
Home^*^	6.23 (0.83)	7.00 (0.00)
School^*^	3.07 (1.80)	6.38 (0.76)
Others^*^	5.15 (1.46)	6.84 (0.37)
**Secondary school**		
Home^*^	5.92 (1.18)	6.92 (0.27)
School^*^	2.76 (1.58)	6.38 (0.50)
Others^*^	4.38 (1.60)	6.76 (0.59)
**At time of testing**		
Home^*^	5.30 (1.49)	6.84 (0.37)
University/Work^*^	2.46 (1.39)	6.46 (0.66)
Others^*^	4.07 (1.03)	5.61 (1.32)
**SELF-RATED PROFICIENCY**		
Global Proficiency^*^	1.00 (0.00)	2.53 (0.66)
Comprehension^*^	1.00 (0.00)	2.38 (0.76)
Speaking^*^	1.23 (0.43)	3.15 (0.89)
Writing^*^	1.38 (0.50)	2.84 (0.89)

*Standard deviations are in parentheses*.

* *Significant differences between early bilinguals and late L2 learners (two-sample t-test comparisons)*.

### Stimuli

Table 1 displays examples of the grammatical and ungrammatical sentences used in the present study. The words in the sentences were all present in the late L2 learners' Basque textbooks. In addition, the grammatical structures tested (verb agreement and ergative case) were early topics in the Basque lessons. Forty grammatical Basque sentences were created. The subject-verb agreement and ergative case violations were derived from the grammatical sentences. Subject-verb agreement violation sentences were created by a mismatch in number between plural subjects and singular verb agreement. Ergative case violation sentences had two arguments with the ergative case. A second grammatical set of sentences was created for comparisons with the object-verb agreement violations. The second set of grammatical sentences was identical to the first set of grammatical sentences except that a plural object agreed with the verb. We created the ungrammatical sentences by changing the grammatical auxiliary verb to a singular object-verb agreement auxiliary.

The experimental sentences were presented with 80 grammatical filler sentences. The critical words were never the last word of the sentence to avoid wrap-up effects. Sentences were digitally recorded at 16-bits by a native, female Basque speaker in a soundproof booth. The sentences across conditions were similar in mean amplitude and length [amplitude: *F*_(6, 234)_ = 1.36, *p* > 0.05; length: *F*_(6, 234)_ < 1].

### Procedure

The ERP recordings were conducted in a soundproof room at the Psycholinguistics Laboratory (University of the Basque Country in Vitoria-Gasteiz). Participants sat in front of a computer monitor in a comfortable armchair. They received written instructions in their L2 (Basque). Participants were instructed to perform a delayed grammatical judgment task (programmed with EXPE6: Pallier et al., 1997). Participants were asked to listen to the sentences and respond whether it was incorrect or correct for each sentence. Responses were given by pushing one of the buttons held in each hand. The correspondence between correct and incorrect responses and hands was counterbalanced across participants. Participants were told about the importance of being still during the ERP recordings. In addition, they were asked to avoid eye movements (including blinking) during the trials. Participants were free to blink between trials when a resting message appeared on the screen. Participants first performed a training of eight practice trials with feedback. For the experiment, participants performed 320 trials. The trials followed a pseudo-random order that did not allow the presentation of more than three successive trials of the same condition. Trials started with a fixation point (an asterisk) for 500 ms, which was then followed by an auditory sentence. Sentences were played binaurally through headphones (Sennheiser HD 435 Manhattan). The asterisk remained on the screen during the full sentence and for 1500 ms after sentence offset. The asterisk was then replaced by a written message that prompted participants to respond. There was no time limit for participants' response. The next trial started 1500 ms after participants' response. A message was presented on the screen during this inter-trial interval that informed participants they could blink freely.

### Electrophysiological recording

The EEG was recorded with the BrainVision 2.0 Analyzer Software package and a BrainAmp amplifier (Brain Products). The EEG signal was recorded from the scalp using tin electrodes mounted in an electro-cap (Electro-Cap International). Electrodes were located at 58 standard positions (Fp2, Fpz, Fp1, F4A, F3A, F2, Fz, F1, F4, F3, F6, F5, F7, F8, C2A, CZA, C1A, C4A, C3A, C6A, C5A, C2, Cz, C1, C4, C3, C6, C5, C2P, C1P, C4P, C3P, T4, T3, T6, T5, T4L, T3L, P2, P1, P6, P5, CB2, CB1, P2P, PZA, P1P, TCP2, TCP1, P4, Pz, P3, P4P, PZP, P3P, O1, Oz, O2). Electrodes attached the outer canthus and to the infra-orbital ridge of the right eye measured eye movements. The EEG recording was referenced online to the right mastoid and re-referenced offline to linked mastoids. Electrode impedances were kept below 5 kΩ. The EEG signal was filtered online with a band-pass between 0.01 and 50 Hz and digitized at a sampling rate of 500 Hz.

### Data analysis

#### Behavioral data

Hit rates were calculated for each participant and condition. Figure 1 displays the global proficiency of the participants, which was calculated by averaging the hit rates for each participant across all conditions. Many late L2 listeners showed poor accuracy levels in the grammatical judgment task. To select those late learners with sufficient accuracy in the grammatical judgment task we compared the hit rates of each late learner for the five conditions against chance level (50%) by means of one-sample *t*-tests. Only those that performed above chance were included in the ERP analysis.

The performance of the selected early bilinguals and late L2 learners was compared for each sentence type (i.e., grammatical, subject-verb agreement violation, ergative case violation, grammatical object and object-verb agreement violation) by means of two-sample *t*-tests on the percentage of hit rates. In addition, the natives' percentage of hit rates was compared to those of early bilinguals and late L2 learners separately by means of two-sample *t*-tests.

#### Electrophysiological data

We used BrainVision Analyzer 2.0 software (Brain Products GmbH, Munich, Germany) to analyze the EEG signal. We used the ocular independent component analyses (Ocular ICA) implemented in BrainVision Analyzer 2.0 Software package (Brain Products) to correct eye movements. We automatically rejected offline those EEG epochs in which any channel either exceeded ±100μV, had an activity below 0.5μV, or showed voltage step/sampling above 50μV within intervals of 200 ms. Both correctly and incorrectly answered trials were included in the analyses to have similar number of epochs for both groups of participants. For the subject- and object-agreement conditions, the epochs were time-locked to the onset of the auxiliary verb in the grammatical and ungrammatical sentences. For the ergative conditions, the epochs were time-locked to the onset of the ergative marker in the second noun in the grammatical and ungrammatical sentences. All epochs included a pre-stimulus baseline of 100 ms and were 1600 ms long. Overall, 10.78% of the trials were rejected from the analysis for the late L2 learners and 6.45% for the early bilinguals. Subsequent independent-samples *t*-tests on the percent of rejected trials for each sentence type separately showed no significant differences between groups (all *p*-values > 0.05). Baseline was corrected and the linear DC Detrend procedure was performed on the individual segments. ERPs were averaged separately for each participant and sentence type.

**Figure 2:**
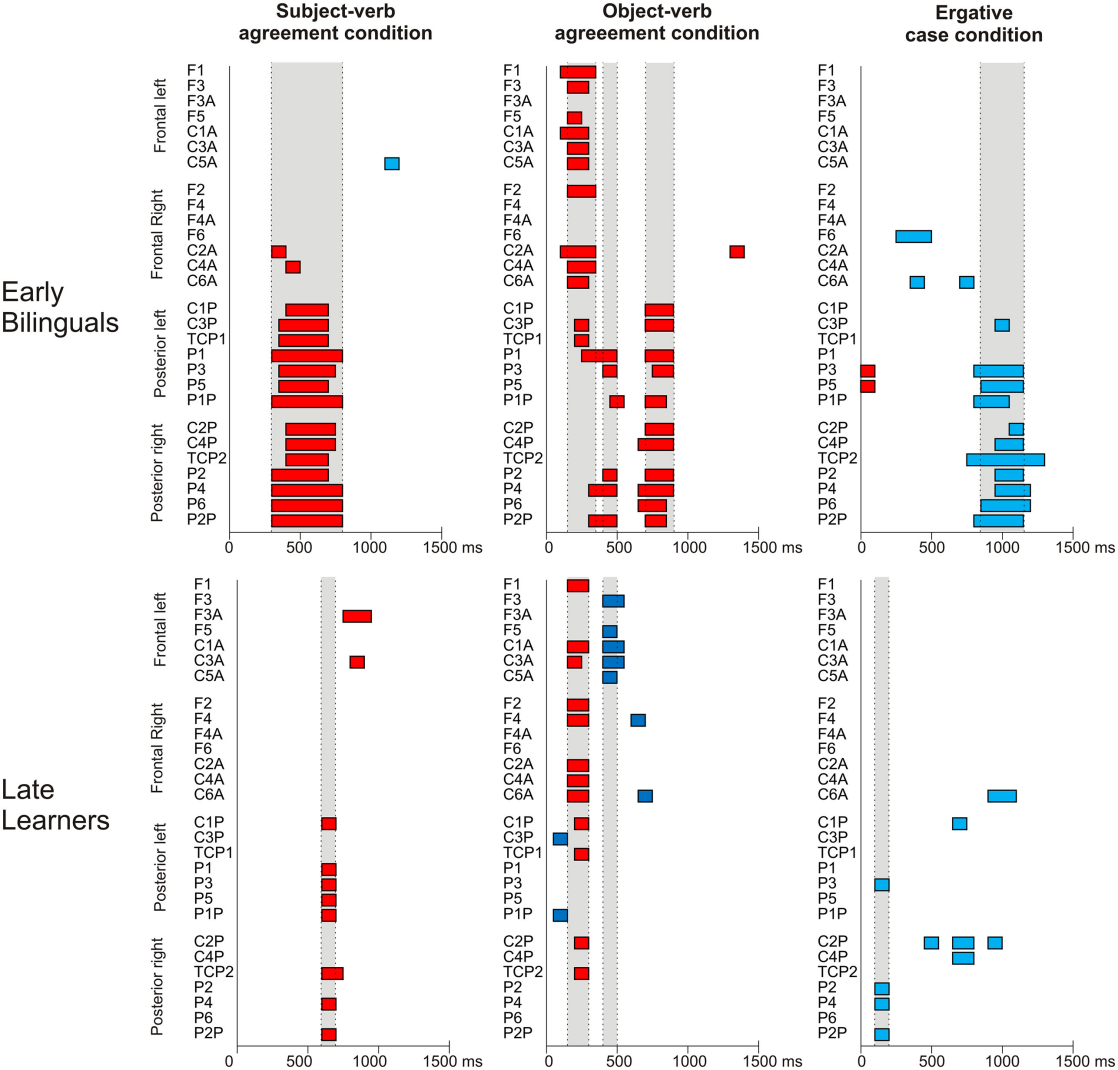
**Results of the *t*-tests on 50-ms consecutive intervals comparing grammatical and ungrammatical sentences at each electrode and for each condition for early and late bilinguals**. The beginning of the epochs are time-locked to the onset of the critical words (i.e., the auxiliary verb for the subject- and object-agreement conditions and the ergative case marker of the second nominal phrase for the ergative case condition). Significant differences between the grammatical and ungrammatical sentences are indicated by the color bars: Red bars correspond to positive effects and blue bars correspond to negative effects. Discontinuous vertical lines mark the onset and offsets of the significant periods. Gray areas indicate the significant time windows.

First, the ERP pattern for each group and condition (subject-verb agreement, object-verb agreement and ergative case) was analyzed separately. We determined the onsets and durations of the ERP effects by means of *t*-tests on 30 successive time windows of 50 ms that compared grammatical and ungrammatical sentences from 0 to 1500 ms at each electrode using Matlab (R2013b, The MathWorks, Inc., MA, USA). Following previous studies, we controlled for false positives that can occur when a large number of statistical comparisons are performed by considering only those effects that were significant in at least two consecutive 50-ms intervals as reliable (Gunter et al., 1997, 2000; Hahne and Friederici, 2001; Díaz et al., 2011). In addition, the onsets and offsets of the effects were set when at least four electrodes showed significant differences between the grammatical and ungrammatical sentences between the given onsets and offsets (Figure 2).

For each condition and significant time window, the groups were compared by means of repeated measures ANOVAs on the mean voltages with the within-subjects factors “Grammaticality,” “Region,” “Hemisphere,” and the between-subjects factor “Bilingual Group.” Effects involving the factor “Grammaticality” (main effect and interactions) and the interaction “Grammaticality” × “Bilingual Group” were of interest. Whenever “Grammaticality” and “Bilingual Group” interacted with “Region” and/or “Hemisphere,” separate ANOVAs were performed to test the interaction of the factors “Grammaticality” × “Bilingual Group” for the particular scalp area. Significance levels of the F-ratios did not need to be adjusted with the Greenhouse-Geisser correction as all main effects and interactions had only one degree of freedom in the numerator. These analyses were performed using IBM SPSS Statistics 19 (SPSS Inc., Chicago, IL, USA).

## Results

### Behavioral data

The global hit score was 80.82% (±17.29%) for the early bilinguals and 66.74% (±13.97%) for the late L2 learners. Both groups showed large individual variability in their accuracy (Figure 1), with the scores ranging from high accuracy (98 and 92% hits for early and late groups, respectively) to very poor (49 and 41% hits for early and late groups, respectively). Ten late learners and five early bilinguals showed very poor performance (below 60% hits). Among late learners, the twelve participants that scored globally above 70% were significantly above chance (*p* < 0.05) and one participant with 69% of hits performed marginally (*p* = 0.069) above chance. These thirteen late L2 learners were considered to have sufficient accuracy levels and were included in the ERP analyses. To match the groups for number of participants, the 13 early bilinguals with the highest global accuracy were included in the ERP analysis.

The subgroup of early bilinguals included in the ERP analysis was more accurate in the grammatical judgment task for all sentences type than the subgroups of late L2 learners (Tables 3, 4). Behaviorally, early bilinguals performed similar to natives (data from Díaz et al., 2011) in all sentence types, although they showed a trend toward poorer performance in the object-verb agreement violations. Late learners performed worse than natives in all sentence types (Tables 3, 4).

**Table 3:** **Mean percentages of correct responses of natives, early bilinguals, and late L2 learners for each experimental condition**.

**Sentence type**	**Natives**	**Early bilinguals**	**Late L2 learners**
Grammatical	95.10 (4.57)	95.38 (3.51)	81.34 (8.39)
Subject-verb agreement violation	91.77 (15.41)	98.07 (2.72)	76.73 (13.78)
Ergative case violation	95.20 (5.04)	95.76 (4.71)	74.23 (7.93)
Grammatical object	93.95 (5.70)	95.00 (5.20)	82.50 (10.30)
Object-verb agreement violation	92.91 (7.46)	88.65 (5.16)	70.00 (15.27)
Total	93.78 (7.63)	94.57 (2.69)	76.96 (6.34)

*Standard deviations are in parentheses. The data from a group of native listeners (Díaz et al., 2011) is shown for the sake of comparison*.

**Table 4:** **Two-sample *t*-test comparisons on the mean percentages of correct responses for each experimental condition**.

**Sentence type**	**Early bilinguals vs. natives**	**Late L2 learners vs. natives**	**Early bilinguals vs. late L2 learners**
Grammatical	*t*_(35)_ < 1	*t*_(35)_ = 6.49, *p* < 0.001	*t*_(24)_ = 5.56, *p* < 0.001
Subject- verb agreement violation	*t*_(35)_ = 1.45, *p* > 0.05	*t*_(35)_ = 2.93, *p* < 0.05	*t*_(24)_ = 8.41, *p* < 0.001
Ergative case violation	*t*_(35)_ < 1	*t*_(35)_ = 9.84, *p* < 0.001	*t*_(24)_ = 5.47, *p* < 0.001
Grammatical object	*t*_(35)_ < 1	*t*_(35)_ = 4.37, *p* < 0.001	*t*_(24)_ = 3.90, *p* < 0.001
Object-verb agreement violation	*t*_(35)_ = 1.82, *p* = 0.076	*t*_(35)_ = 6.16, *p* < 0.001	*t*_(24)_ = 4.17, *p* < 0.001

*All groups of participants were compared to each other in pairs. The data from a group of native listeners (Díaz et al., 2011) are analyzed for the sake of comparison*.

### Electrophysiological data

**Figure 3:**
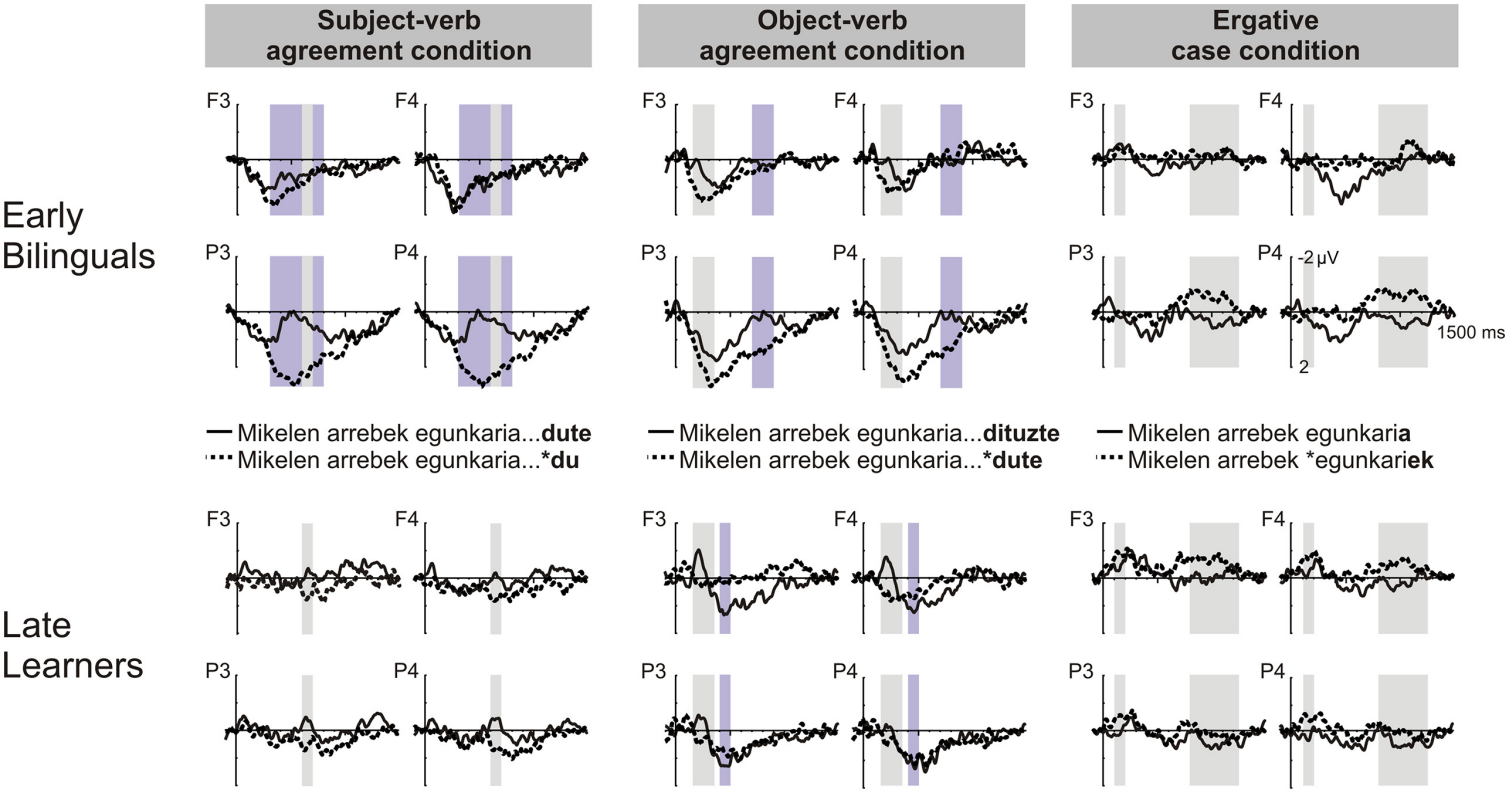
**Grand average waveforms of the early and late groups at four representative electrodes distributed across each scalp area analyzed (frontal right: F4, frontal left: F3, posterior right: P4, posterior left: P3)**. Grand averages are time-locked to the onset of the critical words, i.e., the auxiliary verb for subject- and object-agreement conditions and the morpheme marking the ergative case for the ergative case condition (critical words are depicted in bold in the figure legend). Bars depict the time windows where grammatical and ungrammatical sentences elicited significantly different ERPs. Gray bars depict similar effects between the two groups, and purple bars depict effects which are unique to the given non-native group.

Figure 2 displays the latencies and durations of the ERP effects revealed by the analysis of the 50-ms intervals for each experimental condition and group of participants. Figure 3 displays the grand average waveforms for the two groups of participants and each violation type against the corresponding grammatical condition. In Table 5, significant effects are reported for the ANOVAs run comparing late and early bilinguals with their corresponding *F*- and *p*-values.

**Table 5:** **Effects yielded by the ANOVAs on the mean ERP amplitudes comparing the early and late groups for all three conditions separately and for each significant time window revealed by the 50-ms interval analyses. For the sake of completeness, trends toward significant effects are shown but not further analyzed**.

**Subject-verb agreement condition** [effect: *F*_(1, 12)_]	**Object-verb agreement condition** [effect: *F*_(1, 12)_]	**Ergative case condition** [effect: *F*_(1, 12)_]
*Time window: 600–700 ms* G: 13.01^*^ G × R: 8.61^*^ G × B × R: 6.02^*^ G × B × R × H: 6.55^*^	*Time window: 150–350 ms* G: 14.81^*^Time window: 400–500 ms G × B: 7.80^*^ G × R: 3.34^*^ G × H: 4.36^*^ Time window: 700–900 ms G × B: 4.68^*^ G × R: 5.19^*^	*Time window: 100–200 ms* G: 4.12^+^ G × R: 3.36^#^ *Time window: 800–1150 ms* G: 8.42^*^ G × B × R: 3.62^*^

*G = Grammaticality, R = Region, H = Hemisphere, B = Bilingual Group*.

* *p < 0.05*,

+ *p = 0.053*,

# *p = 0.079*.

#### Subject-verb agreement condition

The statistical comparisons on the 50-ms consecutive windows revealed a positive effect on overlapping time windows, between 300 and 800 ms for early bilinguals and between 600 and 700 ms for late L2 learners (Figure 2). We compared the groups only for the time window in which both groups coincided, i.e., 600–700 ms, to avoid the analysis of the same data in several ANOVAs.

The ANOVA comparing the two groups in the 600–700 ms window showed a significant effect of “Grammaticality,” an interaction between “Grammaticality” × “Bilingual group” × “Region,” an interaction between “Grammatically” and “Region” and an interaction between “Grammaticality” × “Bilingual group” × “Region” × “Hemisphere” (Table 5). Because of the 4-way interaction, further ANOVAs were run with the factors “Grammaticality” and “Bilingual group” separately for each area.

The ANOVAs showed only a significant “Grammaticality” effect in the two posterior areas [posterior left: *F*_(1, 24)_ = 20.01, *p* < 0.001; posterior right: *F*_(1, 24)_ = 24.45, *p* < 0.001]. No effects were significant in the frontal areas. Thus, the posterior positivity elicited by the subject-verb agreement violation in the 600–700 ms window was similar between the groups.

#### Object-verb agreement condition

The analysis on the 50-ms time windows showed a positive effect for both groups between 150 and 350 ms, a positivity for early bilinguals and a negativity for late L2 learners between 400 and 500 ms, and a positivity for early bilinguals between 700 and 900 ms (Figure 2).

The ANOVA comparing the two groups in the 150–350 ms window revealed a significant main effect of “Grammaticality” that did not interact with “Bilingual Group,” “Region,” or “Hemisphere” (Table 5). The grammatical violation elicited a similar broadly distributed positivity in both groups.

In the 400–500 ms window, a significant interaction “Grammaticality” × “Bilingual Group” reached significance, as well as the interactions “Grammaticality” × “Region” and “Grammaticality” × “Hemisphere” (Table 5). The interaction “Grammaticality” × “Bilingual group” was analyzed by running separate ANOVAs for each group. For early bilinguals, no effect was significant. For late L2 learners, there was a significant “Grammaticality” effect [*F*_(1, 12)_ = 8.82, *p* < 0.05] that did not interact with any other factor, hence revealing a broad negativity for ungrammatical sentences.

In the later time window, 700–900 ms, the interactions “Grammaticality” × “Bilingual Group” and “Grammaticality” × “Region” reached significant levels (Table 5). Paired *t*-tests comparing the amplitude for grammatical and ungrammatical sentences separately for each group showed a trend toward a significant “Grammaticality” effect for early bilinguals [*t*_(12)_ = 1.97, *p* = 0.071]. This effect was caused by a broad positivity elicited by the grammatical violations. For late L2 learners, there was no significant effect.

#### Ergative case condition

The analysis on the 50-ms time windows showed a negative effect between 800 and 1150 ms for early bilinguals and between 100 and 200 ms for late L2 learners (Figure 2).

The ANOVAs comparing the two groups in the 100–200 ms window only revealed a trend toward a significant “Grammatical” effect that did not interact with “Bilingual Group” (Table 5). Hence, the effect of grammaticality was reliable for this time window.

The ANOVA comparing the two groups in the 800–1150 ms window showed a main effect of “Grammaticality” and a trend toward a significant interaction “Grammaticality” × “Bilingual group” × “Region.” The triple interaction was analyzed despite being only a trend because it involved the group factor. Further ANOVAs for frontal and posterior regions separately with the factors “Grammaticality” and “Bilingual group” revealed only a main effect of “Grammaticality” in both regions [frontal: *F*_(1, 24)_ = 4.28, *p* < 0.05; posterior: *F*_(1, 24)_ = 11.28, *p* < 0.01] but no “Grammaticality” × “Bilingual group” interaction. The lack of such an interaction showed that both groups displayed similar broad negativities when processing grammatical violations.

## Discussion

The present study compared the brain responses of early bilinguals with high proficiency and late L2 learners with intermediate proficiency (completing a B2 level, Common European Framework) to L2 (Basque) syntactic traits that differed from the participants' L1 (Spanish). The two groups displayed different ERP responses for the agreement conditions but a similar ERP pattern for the ergative condition. As in native listeners (Díaz et al., 2011), subject-verb agreement violations elicited a posterior positivity between 300 and 800 ms in early bilinguals and a short posterior positivity between 600 and 700 ms in late L2 learners. The latency and polarity of the effect coincide with those of the P600, an index of controlled syntactic and reanalysis repair, which has been consistently reported in native listeners for subject-verb agreement violations across many different languages (Coulson et al., 1998a,b; Hahne and Friederici, 1999; Silva-Pereyra and Carreiras, 2007; Díaz et al., 2011). These findings are in agreement with previous studies showing a native-like P600 in response to similar L1–L2 traits for non-native listeners that attained high levels of proficiency, even when the L2 was acquired late in life. Yet, previous studies reported delayed P600 effects for late L2 learners with intermediate proficiency (Rossi et al., 2006; Kotz et al., 2008; Tanner et al., 2013). Thus, the present L2 subject-verb agreement violation triggered syntactic reanalysis and repair processes in both groups of participants but, in the case of late learners, these syntactic reanalysis and repair processes seem to be slower, given the delayed latency of the P600, and shallower, given the short duration of the effect.

For the object-verb agreement violations, both groups displayed a similar broad positivity between 150 and 350 ms that was followed by a broad negativity between 400 and 500 ms in late learners and by a marginally significant positivity between 700 and 900 ms in early bilinguals. Using exactly the same procedures, we previously found an early posterior negativity between 200 and 300 ms and a P600 for object-verb agreement violations in natives (Díaz et al., 2011). This early negativity was interpreted as an N200 component reflecting the violation of phonological expectations. Other studies investigating the processing of object-verb agreement violations in natives have reported an N400-P600 pattern (Zawiszewski and Friederici, 2009; Zawiszewski et al., 2011). The N400 component, classically elicited by unexpected words given a semantic context (Kutas and Federmeier, 2000), has also been showed to index conflicts in thematic role assignment in case violations for languages with overt case marking, such as German, Hindi and Basque (Frisch and Schlesewsky, 2001, 2005; Mueller et al., 2005, 2007; Choudhary et al., 2009; Zawiszewski et al., 2011). Given the association of the N400 to thematic processes, the elicitation of an N400 by object-verb agreement violations was claimed to reflect the establishment of thematic roles during verb agreement computations that involve more than one argument.

Given the previous finding of negativities preceding the P600 for object-verb agreement violations, the early positivity in non-native listeners in the present study was unexpected. However, some studies in native German listeners have reported a positivity, rather than a negativity, for thematic computations at verb agreement processing (Mecklinger et al., 1995; Friederici et al., 1998; Bornkessel et al., 2002, 2003). These studies found a centroposterior positivity between 300 and 400 ms, the so-called P345, time-locked to the onset of auxiliary verbs that disambiguated a relative clause toward an object thematic role. Based on these results, it has been claimed that the function indexed by the P345 is a revision of the thematic role assigned to the arguments (Bornkessel et al., 2002). In line with these studies, the early positivity displayed by non-natives in the present study could be triggered by the revision of the initial assignment of the thematic roles of the noun phrases before syntactic repair and reanalysis processes come into play. The fact that no such positivity was elicited in natives by object-verb agreement violations or in non-natives by subject-verb agreement violations could indicate that thematic computations engender a difficulty in non-natives only when agreement involves the object, the agreement relation missing in their L1. Alternatively, the lack of such a positivity in non-native listeners for subject-agreement processing could indicate that different syntactic computations take place depending on the arguments involved (subject or object), as argued for instance in Zawiszewski and Friederici (2009). This latter interpretation of the results would imply that subject- and object-agreement are two different syntactic phenomena. However, the present data alone is not conclusive as to what the underlying process indexed by the early positivity is.

The early positivity for the object condition was followed by a broad negativity in late learners between 400 and 500 ms rather than a P600. This pattern of results is reminiscent of previous findings with late learners (Osterhout et al., 2006; Guo et al., 2009; McLaughlin et al., 2010; Tanner et al., 2013, 2014). These studies have found an N400, rather than a P600, in novice learners for L2 syntactic violations at the earliest stages of learning but a P600 after 1 year of formal L2 learning in most learners (though there is some individual variation in the timing of the change from an N400 to a P600: Tanner et al., 2014). The N400 in early stages of L2 learning is claimed to index the use of lexical-based heuristics for syntactic processing. The late learners tested in the present study were at early stages of learning. They were in their second year of formal Basque instruction, which makes it likely they are exploiting lexical-semantic aspects rather than syntactic knowledge for accomplishing fast and successful L2 comprehension. In contrast, early bilinguals displayed a marginally significant broad positivity between 700 and 900 ms. We interpret this positivity as an instance of a P600. However, the lack of a posterior distribution and the small amplitude of the positivity suggest that object-verb agreement violations did not trigger native-like reanalysis and repair processes, despite the high proficiency and early AoA of the participants in this group.

This small P600 displayed by early, proficient bilinguals for the object agreement condition contrasts with the native-like P600 effect to object-verb agreement violations reported by Zawiszewski et al. (2011) with a similar population of Spanish early (L1)—Basque (L2) bilinguals who were very proficient in their L2. Three potential sources could be causing, either in isolation or combination, the distinct results reported in Zawiszewski et al. (2011) and the present study. First, the early bilinguals tested in this previous study were similar to natives when detecting object-agreement violations, whereas in the present study, early bilinguals showed a trend toward poorer performance than that of natives in the same task. Second, the use of different sensory modalities for stimuli presentation (written in the previous and auditory in the present study) could be playing a more important role than expected. Despite the fact that sensory modality has been shown not to have an effect on the P600 in native language processing (Hagoort and Brown, 2000; Balconi and Pozzoli, 2005), we cannot rule out that the more demanding auditory presentation, in which word boundaries are not physically present in the stimulus, may be more taxing than visual presentation of isolated words for participants who are not as competent as native listeners. Third, the agreement feature tested in the present and previous study was also different. Zawiszewski et al. (2011) presented person violations, whereas here we presented number violations. It has been shown that subject-person agreement violations engender larger P600 effects than number violations (Nevins et al., 2007; Mancini et al., 2011; Zawiszewski et al., in press). Zawiszewski et al. (in press) studied the Basque native listeners' processing of subject-verb agreement violations of the person feature, number feature, or both. Person violations in all instances elicited a larger P600 than number violations. The same enhancement of the P600 for the person as compared to the number feature has been reported for Hindi and Spanish. Nevins et al. (2007) studied native listeners' processing of subject-verb agreement violations for several features in Hindi: person, number, and gender. A larger P600 was present for feature combinations that encompassed person. Nevins et al. (2007) concluded that the person feature has a greater salience than other features. Similarly, Mancini et al. (2011) also found a larger and more broadly distributed P600 for person than for number subject-verb agreement violations in Spanish. This difference in the salience of the person and number features agreement may explain why the present group of early bilinguals displays a small P600 effect, while a native-like P600 was reported by Zawiszewski et al. (2011).

The ergative case violation elicited the same brain responses in both groups of non-native listeners: a broad negativity between 800 and 1150 ms. Therefore, neither AoA nor proficiency modulates the ERP responses. The ERP pattern displayed by non-natives was qualitatively different than that of native listeners, who displayed the typical P600 (Díaz et al., 2011). The absence of a P600 for the non-native groups is in line with the findings in Zawiszewski et al. (2011). They found an N400-P600 pattern in natives but only an N400 in early, proficient bilinguals. The N400 preceding the P600 (Frisch and Schlesewsky, 2001, 2005; Mueller et al., 2005, 2007; Zawiszewski et al., 2011) or in isolation (Choudhary et al., 2009) has been repeatedly reported for case violations and is interpreted as indexing processes of thematic assignment. The present negativity does not possess the typical N400 latency (peaks at around 400 ms) or anterior scalp distribution for auditory sentence presentation (Holcomb and Neville, 1990; Connolly and Phillips, 1994; Mueller et al., 2005). However, it is possible that, in non-native listeners, the latency and topography of the ERP components, frequently delayed and broadly distributed as reported for the P600 (Weber-Fox and Neville, 1996; Hahne, 2001; Zawiszewski et al., 2011), does not correspond to those elicited by native listeners. It remains unclear whether the function of the present negativity is analogous to the one reported in natives, which is associated to thematic assignment processes (Frisch and Schlesewsky, 2001, 2005; Mueller et al., 2005, 2007; Choudhary et al., 2009; Zawiszewski et al., 2011), or to the one reported in novice learners instead of P600s, which indexes the use of lexical heuristics (Osterhout et al., 2006; Guo et al., 2009; McLaughlin et al., 2010; Tanner et al., 2013, 2014). We favor the latter interpretation, given that the group of natives tested with the same procedures did not display an N400 (Díaz et al., 2011).

One limitation of the present study is the small sample size, given the very low proficiency in the experimental task of several late learners. The sample size might reduce the sensitivity when trying to capture differences between the groups. Nevertheless, we were able to assess reliable ERP effects within and between the groups. Our results suggest that processing L1–L2 similar traits, like verb-agreement, engages native-like responses (i.e., a P600) in highly proficient, early bilinguals. We interpret this pattern of results as an indication that the presence of verb agreement in the participants' L1 allows verb agreement in L2 to be processed in a native-like fashion, independently of which core arguments are involved in the agreement relation, when the L2 is learned early in life and high proficiency is attained (Zawiszewski et al., 2011). The reduced P600 displayed by proficient, early bilinguals for the object agreement condition together with the non-native early positivity suggests an increased difficulty in applying the L1 processing routines of subject-verb agreement to the processing of the L2 object-agreement. The increased difficulty in processing object agreement, as compared to subject-verb agreement, is further corroborated by the lack of a P600 effect in late L2 learners. Overall, for the agreement conditions, highly proficient, early bilinguals approximated native processing to a greater extent than late learners with intermediate proficiency. This suggests that the processing of L1–L2 converging traits is influenced by AoA and proficiency. However, the L2-only trait, the ergative case condition, elicited a similar response in both L2 groups: a delayed and broad N400 that was qualitatively different to that of natives, i.e., a P600. This finding indicates that neither AoA nor proficiency influences the brain responses to syntactic traits that are unique to L2. Thus, the comparison of the results between the non-native groups and across the different grammatical traits tested suggests that L1-L2 similarity plays a major role in the neural mechanisms engaged in L2 syntactic processing. The computation of L2 syntactic dependencies engages neural mechanisms that are already present in L1 processing, and the degree to which the pre-existing L1 neural routines can be successfully exploited in the processing of the L2 is influenced by AoA and/or proficiency. In sharp contrast, the processing of syntactic traits that are unique to L2 requires the implementation of new neural routines which do not depend on the L2 age of acquisition, at least when sufficient proficiency is attained in the L2. Nevertheless, the underlying neural processes do not seem to involve native-like processes, even in case of early AoA and high proficiency. Future studies comparing other language pairs are needed to evaluate the cross-linguistic validity of the present findings.

## Author contributions

All authors listed have made substantial, direct, and intellectual contribution to the work and have approved it for publication.

### Conflict of interest statement

The authors declare that the research was conducted in the absence of any commercial or financial relationships that could be construed as a potential conflict of interest. The reviewer RT and the handling Editor declared their shared affiliation, and the handling Editor states that the process nevertheless met the standards of a fair and objective review.

## Footnotes

^1^The present study does not present a direct comparison of the natives and non-natives data sets because of differences in the number of participants for each group (24 native listeners were tested in the previous study vs. 13 participants in each non-native group) and differences in the analysis procedures (natives' brain responses were analyzed with EEprobe (ANT, The Netherlands) and no eye correction was applied, whereas non-natives' responses were analyzed with BrainVision Analyzer 2.0 (Brain Products GmbH, Munich, Germany) and eye movements were corrected.
